# Coinfection of porcine deltacoronavirus and porcine epidemic diarrhea virus increases disease severity, cell trophism and earlier upregulation of IFN-α and IL12

**DOI:** 10.1038/s41598-021-82738-8

**Published:** 2021-02-04

**Authors:** Kepalee Saeng-chuto, Adthakorn Madapong, Kampon Kaeoket, Pablo Enrique Piñeyro, Angkana Tantituvanont, Dachrit Nilubol

**Affiliations:** 1 Department of Veterinary Microbiology, Faculty of Veterinary Science, Chulalongkorn University, Henry Dunant Road, Pathumwan, Bangkok, 10330 Thailand; 2 Department of Clinical Sciences and Public Health, Faculty of Veterinary Science, Mahidol University, Nakhon Pathom, Thailand; 3 Department of Veterinary Diagnostic and Production Animal Medicine, College of Veterinary Medicine, Iowa State University, Ames, IA USA; 4 Department of Pharmaceutics and Industrial Pharmacy, Faculty of Pharmaceutical Sciences, Chulalongkorn University, Bangkok, Thailand; 5 Cell-Based Drug and Health Product Development Research Unit, Faculty of Pharmaceutical Sciences, Chulalongkorn University, Bangkok, Thailand

**Keywords:** Immunology, Microbiology, Pathogenesis

## Abstract

Porcine epidemic diarrhea virus (PEDV) and porcine deltacoronavirus (PDCoV) cause an enteric disease characterized by diarrhea clinically indistinguishable. Both viruses are simultaneously detected in clinical cases, but a study involving the co-infection has not been reported. The study was therefore conducted to investigate the disease severity following a co-infection with PEDV and PDCoV. In the study, 4-day-old pigs were orally inoculated with PEDV and PDCoV, either alone or in combination. Following challenge, fecal score was monitored on a daily basis. Fecal swabs were collected and assayed for the presence of viruses. Three pigs per group were necropsied at 3 and 5 days post inoculation (dpi). Microscopic lesions and villous height to crypt depth (VH:CD) ratio, together with the presence of PEDV and PDCoV antigens, were evaluated in small intestinal tissues. Expressions of interferon alpha (IFN-α) and interleukin 12 (IL12) were investigated in small intestinal mucosa. The findings indicated that coinoculation increased the disease severity, demonstrated by significantly prolonged fecal score and virus shedding and decreasing VH:CD ratio in the jejunum compared with pigs inoculated with either PEDV or PDCoV alone. Notably, in single-inoculated groups, PEDV and PDCoV antigens were detected only in villous enterocytes wile in the coinoculated group, PDCoV antigen was detected in both villous enterocytes and crypts. IFN-α and IL12 were significantly up-regulated in coinoculated groups in comparison with single-inoculated groups. In conclusion, co-infection with PEDV and PDCoV exacerbate clinical signs and have a synergetic on the regulatory effect inflammatory cytokines compared to a single infection with either virus.

## Introduction

Porcine epidemic diarrhea virus (PEDV) and porcine deltacoronavirus (PDCoV) are 2 of the 3 viruses in the family *Coronaviridae*^1, 2^ that have presently continued to cause economic disadvantages to the swine industry worldwide, especially in Asia. PEDV and PDCoV are enveloped, positive-sense, single-stranded RNA viruses that belong to different genera but the same family *Coronaviridae* and order *Nidovirales*^3^. PEDV belongs to the genus *Alphacoronavirus*, while PDCoV belongs to the genus *Deltacoronavirus*. Both viruses induce similar pathogenesis, including transmission through the fecal–oral route, and primarily infect villous enterocytes of the small intestine. This leads to indistinguishable clinical signs characterized by malabsorptive diarrhea, dehydration, vomiting, and high mortality^4, 5^. Pigs at all ages are susceptible to both viruses, but mortality is high in pigs under one week of age. Mortality rates in PEDV-infected piglets are higher than those in PDCoV-infected piglets^1^.

PEDV and PDCoV have a different genome characteristic. The full-length genome of PEDV is approximately 28 kb in length, consisting of open reading frames (ORF) 1a and 1b, spike (S), ORF3, envelop (E), membrane (M), and nucleocapsid (N), and flanked by 5′- and 3′-untranslated regions (UTR)^6^. In contrast, the full-length genome of PDCoV is approximately 25 kb in length, comprising ORF1a/1b, S, E, M, N, nonstructural protein 6 (Nsp6), Nsp7, and flanked by 5′- and 3′-UTRs^7^. Despite their genome organization differences, the S gene, encoding S glycoprotein, plays an essential role in both viruses’ pathogenesis. The S glycoprotein has two domains that accomplish two main functions, including the S1 domain important for host-cell receptor binding and the S2 domain that functions in entry into host cells by cell membrane fusion^8, 9^.

PEDV was first reported in 1976–1978 in Belgium and the United Kingdom^2, 10^. Following its emergence, PEDV became endemic, especially in Asia^11–14^. Presently, 2 genogroups of PEDV, genogroups 1 and 2, have been recognized^15, 16^. Each genogroup further evolved into 2 subgroups, including 1a and 1b and 2a and 2b. The cluster system is based mainly on the genetic diversity of the S gene region. The G2 genogroup contains two insertions of 4 (^56^GENQ^59^) and 1 (^140^N) amino acids at positions 55–60 and 140, respectively, and a deletion of 2 amino acids (^160^DG^161^) at positions 160–161. In Thailand, a PED outbreak was first observed in 2007; PEDV genogroup 2 was responsible for the outbreak. PEDV genogroup 1 was detected later in 2013 due to illegally smuggled modified live vaccine of genogroup 1^17^. Presently, PEDV genogroup 2 is considered the main genogroup is causing economic losses to the Thai swine industry^13^, and its evolutionary rate is high^18^.

PDCoV was first reported in Hong Kong in 2012, without clinical evidence of disease^7^. The evidence of PDCoV causing the clinical disease was first reported in pigs in Ohio, US, followed by 18 other states in the US in 2014^1^. Soon after its emergence in the US, PDCoV was reported in several countries including China, South Korea, Thailand, Lao PDR, and Vietnam in 2015^19–25^. Presently, 3 groups, including the US, China, and Southeast Asia (SEA) clusters, have been recognized^21, 22, 26^. Previous study reported that up to 51% of swine herds with coronavirus diarrhea showed a PEDV and PDCoV co-infection^22, 26–29^. Whether the co-infection would exacerbate clinical diseases remains unknown.

The innate immune system is the first line of defense against viral infection. Interferons (IFNs) were firstly reported in 1957 as soluble glycoproteins with strong antiviral effects^30, 31^. IFNs are divided into three types (types I, II, and III) based on their sequence similarity, cell-surface receptors, and biological function^32^. Type I IFNs, such as IFN-α and IFN-β, are recognized to inhibit viral replication and mediate protection against viral infection^33^. IFN-α is a pluripotent inflammatory cytokine naturally induced by viral infections. Interleukin 12 (IL12), an innate cytokine produced by macrophages and dendritic cells, which can be stimulated during viral infections, is supposed to be responsible for improving Type 1 T helper (Th1) cells and secretory immunoglobulin A (SIgA) response at the mucosal level^34^.The expression of cytokines in small intestinal mucosa of piglets single-infected with PEDV or PDCoV were recently reported^35, 36^. Previous study demonstrated that PDCoV infection significantly induced type I IFN production^36^. Moreover, infection of PEDV non-S-INDEL lead to suppression of IFN-α, while PEDV S-INDEL infection lead to up-regulation of IL12^35^.

PEDV and PDCoV continue to cause severe economic losses on swine farms, and frequent outbreaks are observed despite intensive control regimens. The unsuccessful control could be due to several factors, including ineffective immunization methods such as vaccination and feedback and misdiagnosis between PEDV and PDCoV infections. Besides, co-infection with both PEDV and PDCoV could increase the severity of clinical diseases. Although severity of infection with PEDV or PDCoV has been described in many previous studies, severity of co-infection with these two viruses has not been reported^4, 37, 38^. Therefore, this study was conducted to investigate disease severity in pigs inoculated with PED and/or PDCoV. Diarrhea severity, virus shedding, villous height, crypt depth (VH:CD) ratio, and viral distribution in the small intestine were compared. In this study, not only disease severity was investigated, the expression of IFN-α and IL12 were evaluated in the small intestinal mucosa of piglets either single- or co-inoculated with PEDV and PDCoV.

## Materials and methods

### Virus isolates and propagation

PDCoV isolates NT1_1215 (accession number KX361345) and PEDV isolate P1915-NPF-071511A (accession number KX981900) were used in the study. These two viruses were isolated from two pig herds experiencing PDCoV and PEDV outbreaks.

LLC-PK1 cells (ATCC CL-101) and Vero C1008 cells (ATCC CRL-1586) were used to propagate PDCoV and PEDV, respectively. Vero C1008 and LLC-PK1 cells were maintained using growth medium (Dulbecco’s Modified Eagle Medium (DMEM; Gibco, USA) supplemented with 10% heat-inactivated fetal bovine serum (FBS; Gibco, USA) for virus propagation. At 80% confluency, growth medium was discarded, and the cells were washed twice with 1X PBS (1X phosphate-buffered saline; 0.1 M, pH 7.2) followed by maintenance medium (DMEM (Gibco, USA) supplemented with 8 µg/ml trypsin/EDTA (Gibco, USA)). Each virus was added into each cell line and incubated at 37 °C with 5% CO_2_ for 60 min. After incubation, the inoculated cells were washed twice with 1X PBS. A maintenance medium was added to the inoculated cells, and the cells were incubated at 37 °C with 5% CO_2_ until a cytopathic effect (CPE) was observed.

### Ethical statement for experimental procedures

All animal procedures were performed following the Guide for the Care and Use of Laboratory Animals of the National Research Council of Thailand according to protocols reviewed and approved by the Faculty of Veterinary Science, Mahidol University-Institute Animal Care and Use Committee (FVS-MU-IACUC; animal use license number U1-01281-2558).

### Experimental design

Twenty-four 4-day-old piglets were procured from a herd that had not a history of PEDV or PDCoV outbreaks. The negative status of PEDV, PDCoV, TGEV, and porcine rotavirus (groups A, B, and C) was confirmed by virus-specific RT-PCR on rectal swabs. Upon arrival, all pigs were randomly allocated into 4 groups including (G1) PDCoV-inoculated group (n = 6), (G2) PEDV-inoculated group (n = 6), (G3) co-inoculated group (n = 6), and (G4) control group (n = 6). All piglets in G1 and G2 were inoculated orally with 5 ml of each virus at a titer of 10^3^ TCID_50_/ml. Piglets in G3 were inoculated orally with 5 ml of a mixture of both viruses (2.5 ml PDCoV and 2.5 ml PEDV) at a titer of 10^3^ TCID_50_/ml. Piglets in G4 were inoculated orally with 5 ml of a mock control.

Piglets were observed daily for clinical signs, including vomiting, diarrhea, lethargy, and body condition. Fecal score was evaluated based on the following criteria: 0 = normal, 1 = soft (cowpie), 2 = mild or liquid with some solid content, 3 = severe or liquid with no solid content.

Three piglets in each group were euthanized at 3- and 5-days post-inoculation (dpi). At necropsy, the small intestine, cecum, and colon were examined for the presence of gross lesions. The small intestine, including the duodenum, proximal jejunum, middle jejunum, distal jejunum, and ileum, were collected and fixed in 10% formalin for further histological evaluation by hematoxylin and eosin (H&E) staining and specific PEDV and PDCoV immunohistochemistry (IHC). Five mg of intestinal mucosa was collected by scraping with a sterile scalpel blade and kept into RNAlater Stabilization Solution (Life Technologies, Carlsbad, CA) to evaluate the IFN-α and IL12 gene expression.

### Cloning and plasmid construction

Viral RNA was extracted from each propagated virus using a Nucleospin Viral RNA Extraction Kit (Macherey-Nagel Inc., PA, USA) and then converted to cDNA using M-MuLV Reverse Transcriptase (New England BioLabs Inc., MA, USA). The PEDV N gene was amplified using specific primers as previously reported^39^. For PDCoV N gene amplification, specific forward and reverse primers were designed and named PDCoV_qPCR_KS_F (5′-TGGCAATGGAGTTCCGCTTA-3′) and PDCoV_qPCR_KS_R (5′-GGGTATCATTAGGAGGGAGTT-3′), respectively. The PCR was performed using 2 × PCR Master Mix Solution *(i-Taq*) (iNtRON Biotechnology Inc., Seongnam-Si, Korea). The PCR products were electrophoresed at 100 V for 30 min on a 1% agarose gel before the gel was stained with RedSafe nucleic acid staining solution (iNtRON Biotechnology Inc., Seongnam-Si, Korea) and examined under a UV light. Bands of target genes were purified using the Nucleospin Plasmid kit (Macherey–Nagel Inc., Bethlehem, PA, USA) and cloned into pGEM-T Easy Vector systems (Promega, Madison, WI, USA). Recombinant plasmids were transformed into competent *E. coli* according to a previously described method^13^. The transformed *E. coli* cells were spread on Luria–Bertani (LB) agar plates supplemented with 100 µg/ml ampicillin, incubated at 37 °C overnight, then checked by colony PCR with the specific primers. A colony of each virus that contained each target gene was scaled up. The plasmids were extracted using a Nucleospin Plasmid kit (Macherey-Nagel Inc., PA, USA) and used to generate standard curves in quantitative PCR (qPCR).

### Viral shedding

One mg feces were collected at 0, 3, and 5 dpi by inserting a sterile cotton swab into the rectum. The swabs were kept in 1 ml RNAlater Stabilization Solution (Life Technologies, Carlsbad, CA). Viral RNA was extracted from fecal samples using the Nucleospin Viral RNA Extraction Kit (Macherey-Nagel Inc., PA, USA), then converted to cDNA using M-MuLV Reverse Transcriptase (New England BioLabs Inc., MA, USA). The PEDV shedding was determined using qPCR with specific primers and probes as previously reported^39^, while PDCoV shedding was determined with the designed primers (PDCoV_qPCR_KS_F and PDCoV_qPCR_KS_R) and the PDCoV_qPCR_KS_P probe (5′-FAM-TGGCACAGGTCCCAGAGGAAATCT-BHQ1-3′). The qPCR reaction was performed using Maxima Probe/ROX qPCR Master Mix (2X) (Thermo Scientific, Pittsburgh, PA) in the QuantStudio 3D Digital PCR System (Applied Biosystems, Waltham, MA. Each sample was run in triplicate.

### Small intestine villous height and crypt depth (VH:CD) ratio

The villous height and crypt depth (VH:CD) ratio were evaluated light microscopy on histological sections stained by hematoxylin and eosin (H&E). Multiple sections from different anatomic locations of small intestine previously fixed in 10% neutral buffered formalin were dehydrated, impregnated with, and embedded in paraffin, sectioned at 5 μm and mounted on glass slides. Slides with tissue sections were incubated at 60 °C for 30 min, and deparaffinization was completed by slide immersion in xylenes and then rehydration in absolute alcohol and water, respectively. The slides were stained with hematoxylin, followed by destaining with 1% hydrochloric acid ethanol, and stained with eosin. Finally, the slides were dehydrated, cleared with xylene, and coverslipped. Villous height and crypt depth (VH:CD) ratios were estimated by measuring 10 villi and crypts throughout the section and calculated using the NIH ImageJ 1.50i Program (http://rsb.info.nih.gov/ij).

### Immunohistochemistry (IHC)

Tissue sectioning and slide preparation follow the same protocol previously described for H&E staining. Antigen retrieval was performed using proteinase K (Invitrogen, Grand Island, NY, USA). Endogenous peroxidases and background were blocked using hydrogen peroxide (Sigma-Aldrich, Steinheim, Germany) and goat serum supplemented with Triton X-100 (Bio-Rad Laboratories, Hercules, CA, USA), respectively. Slides were incubated with anti-N PEDV or PDCoV antibody (Medgene Labs, Brooking, SD) at a dilution of 1:1,000 as the primary antibody, followed by the Dako REAL EnVision/HRP detection system, with rabbit/mouse (ENV) (Dako, Copenhagen, Denmark) as a secondary antibody. All slides were treated with the Dako Real EnVision Detection System, Peroxidase/DAB + , rabbit/mouse (Dako, Copenhagen, Denmark). The slides were then counterstained with Mayer’s hematoxylin (Sigma-Aldrich, St. Louis, MO, USA) before dehydration, clearing with xylene, and coverslipped. Sections of the small intestine from negative control animals were used as a negative control.

IHC scoring was recorded as follows: 0 = No staining, 1 = 1–10% enterocytes with positive staining, 2 = 11–25% enterocytes with positive staining, 3 = 26–50% enterocytes with positive staining, 4 = 50–100% enterocytes with positive staining^37, 38^. The IHC score was calculated by measuring 10 fields throughout the section and calculated using the NIH ImageJ 1.50i (Fiji) Program (http://rsb.info.nih.gov/ij).

### Expression of IFN-α and IL12 in the small intestinal mucosa

RNA was extracted from five mg of intestinal mucosa using Qiagen RNeasy Plus mini kit (Qiagen; Hilden, Germany). Two µg of RNA was converted to cDNA using M-MuLV Reverse Transcriptase (New England BioLabs Inc., MA, USA). cDNA was used to evaluate the gene expression of IFN-α and IL12 using qPCR with specific primer pairs as previously reported^35^. The qPCR reaction was performed using Maxima SYBR Green/ROX qPCR Master Mix (2X) (Thermo-Fisher Scientific, MA, USA) in the QuantStudio 3D Digital PCR System (Applied Biosystems, Waltham, MA, USA). Each sample was run in triplicate. Relative expressions were evaluated using the 2^−ΔΔCt^ method according to previously reported^40^. Glyceraldehyde-3-phosphate dehydrogenase (GAPDH) and beta-actin were used as an internal control to normalize changes in specific gene expressions. The results were presented as fold changes relative to the control animals.

### Statistical analysis

The fecal score, viral shedding, VH:CD ratio, IHC score, and the fold changes in mRNA expression were compared using one-way analysis of variance, followed by Tukey’s multiple comparison test implemented in GraphPad Prism 7 (GraphPad Software Inc., La Jolla, CA, USA) and IBM SPSS Statistics version 22 (IBM Corp., Armonk, NY, USA) was used in the data analysis. *p* < 0.05 was considered significant.

## Results

### PEDV and PDCoV co-inoculation increased the severity of clinical diarrhea

**Figure 1:**
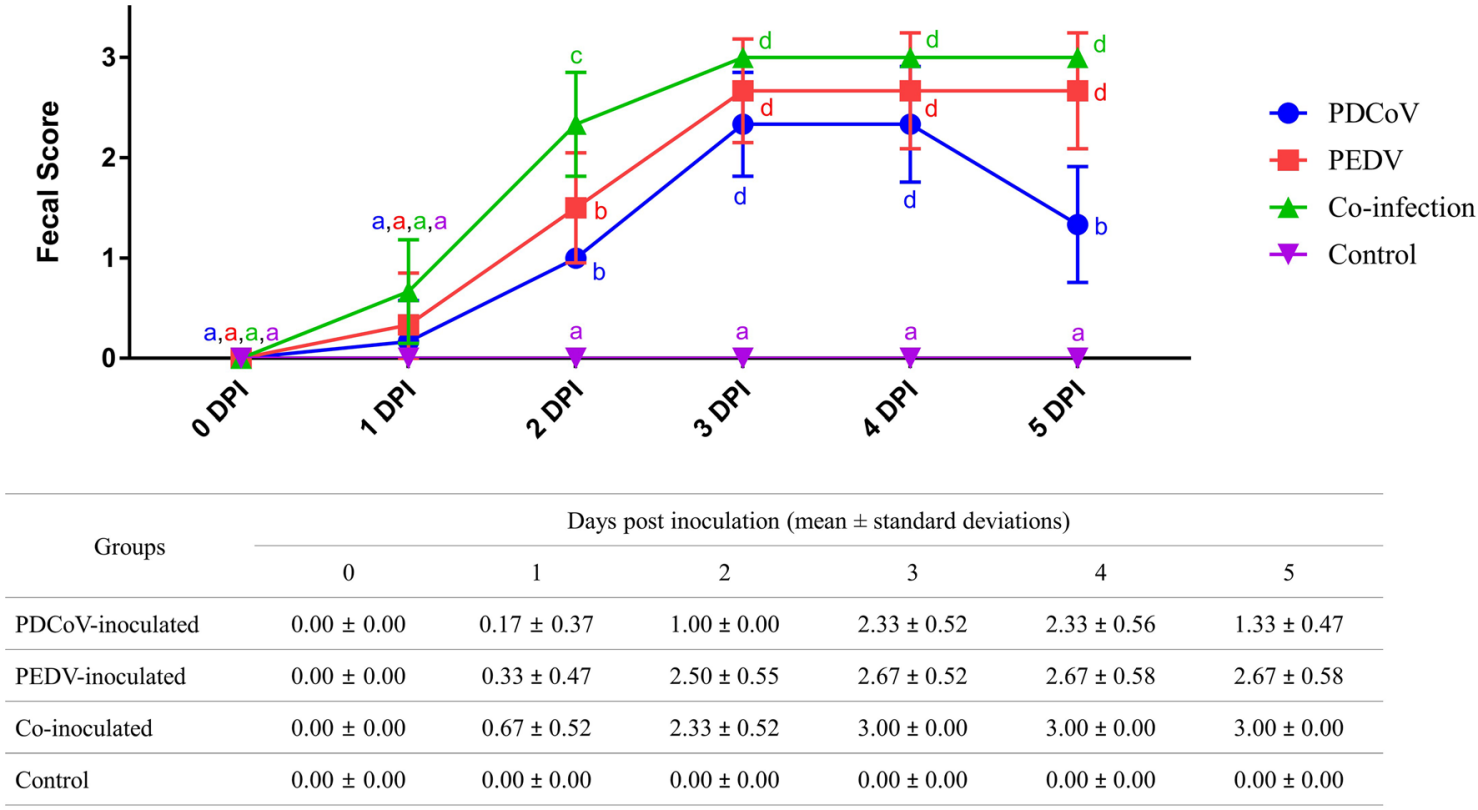
Fecal scores of piglets at 0- to 5-days post-inoculation (dpi). Blue circles, red squares, green triangles, and purple triangles represent PDCoV-inoculated, PEDV-inoculated, co-inoculated, and control groups. Different lower-case letters indicate significant differences between each group and each dpi (*p* < 0.05). Values of mean ± standard deviation (SD) of each group, and each dpi are presented in a table under the graph.

The dynamic of the fecal score for each inoculated group is shown in Fig. 1. Piglets in all inoculated groups showed similar fecal score severity trends, which slowly increased from 1 dpi, reaching a maximum score by 3 dpi. The co-inoculated group had a higher proportion of clinically affected animals (4 out 6), compared to PDCoV (1 out 6)- and PEDV-inoculated (2 out 6) groups, respectively. At 2 dpi, 4 out of 6 piglets of the co-inoculated group had mild, and 2 out of 6 piglets had severe diarrhea. At 3–5 dpi, all piglets in the co-inoculated group had severe diarrhea. In contrast, all piglets developed soft feces at 2 dpi before developing mild (4 out of 6 piglets) and severe (2 out of 6 piglets) diarrhea at 3 dpi in the single PDCoV inoculated group. Three out of 3 pigs in the PEDV-inoculated group progressed to soft and mild diarrhea, respectively, at 2 dpi. At 3 dpi, the average fecal score of all 6 piglets in the PEDV-inoculated group was significantly higher. The severity of the fecal score of the PDCoV-inoculated group decreased from 3 to 5 dpi and displayed signs of recovery. In contrast, in the PEDV- and co-inoculated groups, the fecal scores’ severity at 3 and 5 dpi remained at the same level. Interestingly, co-inoculated piglets showed more severe diarrhea (3 out of 3 piglets) than the single PED-inoculated group (2 out of 3 piglets). Neither animals in the PEDV- and co-inoculated groups showed recovery signs by the end of the study.

### Coinoculation significantly increased PEDV and PDCoV shedding

**Figure 2:**
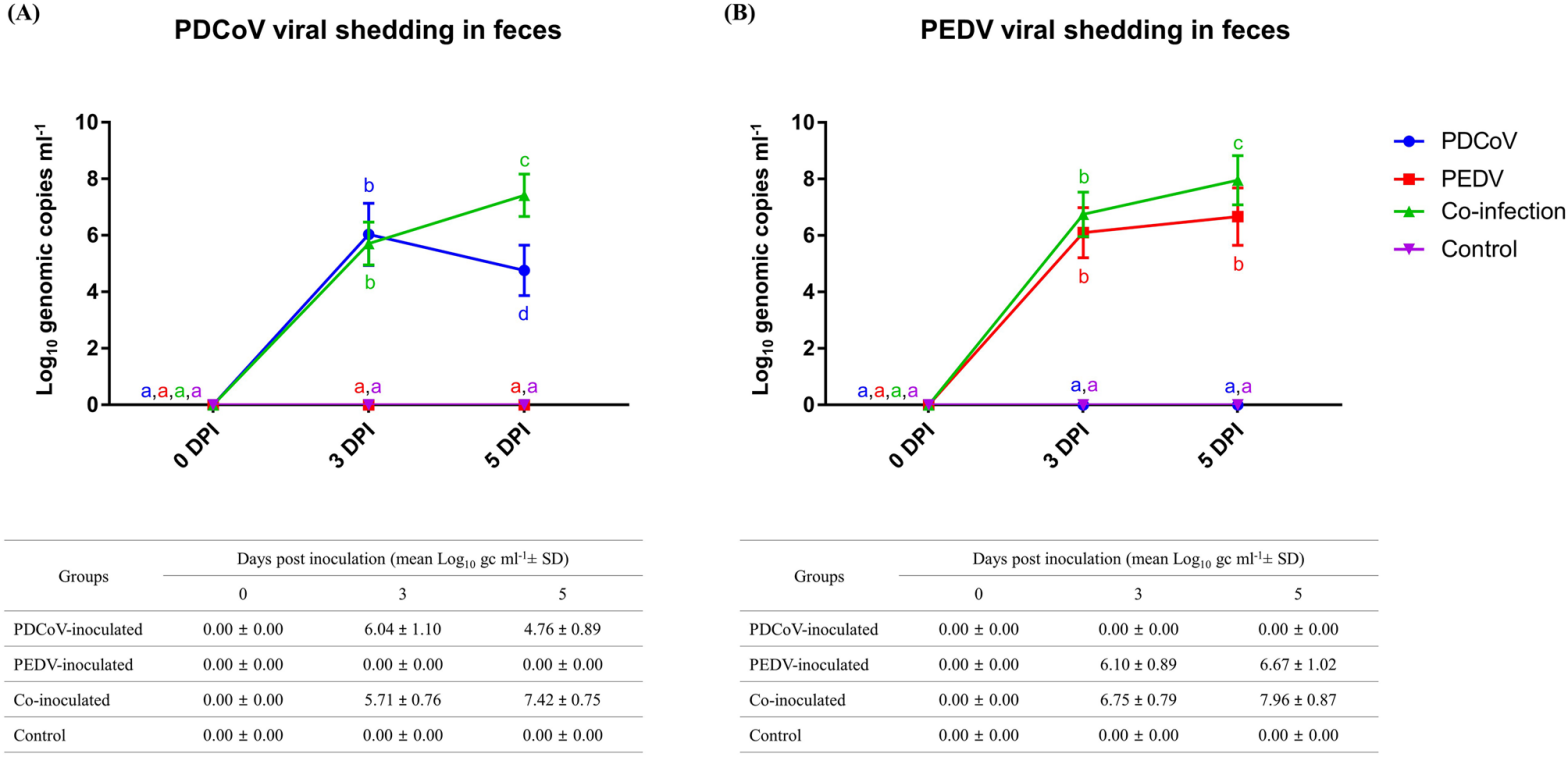
PDCoV (**A**) and PEDV (**B**) shedding in rectal swabs at 0-, 3- and 5-days post-inoculation (dpi). Blue circles, red squares, green triangles, and purple triangles represent PDCoV-inoculated, PEDV-inoculated, co-inoculated, and control groups. Different lower-case letters indicate significant differences between each group and each dpi (*p* < 0.05). Values of mean ± standard deviation (SD) of each group, and each dpi are presented in a table under the graph.

PDCoV and PEDV shedding detected by qPCR in fecal swab samples is presented in Fig. 2. At 3 dpi, the PDCoV shedding level was not significantly different between the PDCoV-inoculated and co-inoculated groups. However, at 5 dpi, the PDCoV shedding level was significantly higher in the co-inoculated group due to a significant shedding reduction in the PDCoV-inoculated group (Fig. 2A). Like the PDCoV shedding pattern, the PEDV shedding levels were not significantly different between the PEDV-inoculated and co-inoculated groups at 3 dpi. The PEDV shedding levels did not change significantly in the PEDV-inoculated group between 3- and 5 dpi. However, there was a significant increase in the co-inoculated group compared with the PEDV-inoculated group at 5 dpi (Fig. 2B).

### Coinoculated groups had significantly lower VH:CD ratios than single-inoculated groups

**Figure 3:**
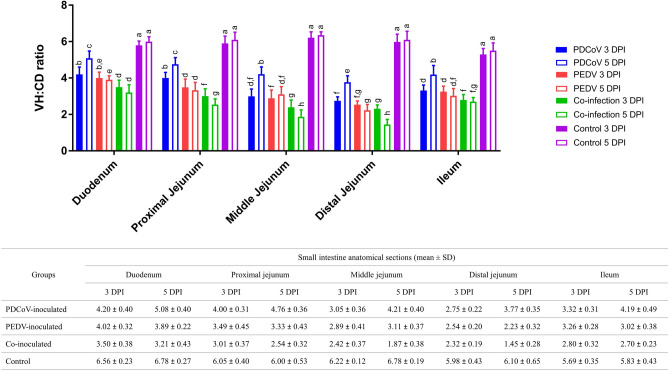
Villous height and crypt depth (VH:CD) ratio in the duodenum, proximal jejunum, middle jejunum, distal jejunum, and ileum. Blue, red, green, and purple bars represent PDCoV-inoculated, PEDV-inoculated, co-inoculated, and control groups. Solid and open bars represent 3- and 5-days post-inoculation (dpi), respectively. Different lower-case letters indicate significant differences between each group, each dpi, and each anatomical section of the small intestine (*p* < 0.05). Values of mean ± standard deviation (SD) of each group, and each dpi are presented in a table under the graph.

The VH:CD ratios of piglets in single-infected and co-infected groups are shown in Fig. 3. All inoculated groups had significantly lower VH:CD ratios in all small intestine regions compared with the control group. The co-inoculated groups had significantly lower VH:CD ratios compared with each individual inoculated group. The PDCoV-infected group had a significantly higher VH:CD ratio than the other 2 groups suggesting that PDCoV induces milder enteric changes compared to the PEDV-infected group. It is interesting to note that, following inoculation, the VH:CD ratio of the middle and distal jejunum was the lowest than those in the other regions. The shorten villi primarily observed at the middle and distal jejunum suggested that these tissues could serve as the primary target tissue. Meanwhile, the VH:CD ratio of the duodenum was the highest compared to the other intestinal regions evaluated.

The co-inoculated group showed a significantly lower VH:CD ratio at 3 and 5 dpi than the single-inoculated groups in the proximal jejunum. Also, the VH:CD ratio of the co-inoculated group was significantly shorter at 5 dpi than 3 dpi. The VH:CD ratio of the PDCoV-inoculated group was significantly higher than the PEDV-inoculated group at 3- and 5-dpi. The VH:CD ratio of the PDCoV-inoculated piglets was significantly higher, 5 dpi compared to 3 dpi.

In the middle jejunum, the VH:CD ratio of the single-inoculated groups was significantly higher than the co-inoculated group at 3 and 5 dpi. Although the VH:CD ratio between the PDCoV- and PEDV-inoculated groups was not different at 3 dpi, it was significantly higher in the PDCoV-inoculated group compared to PEDV-inoculated at 5 dpi. The VH:CD ratio of the PDCoV-inoculated group was significantly higher at 5 dpi compared to 3 dpi. In contrast, the VH:CD ratio of the co-inoculated group was significantly lower at 5 dpi than 3 dpi.

The VH:CD ratios were not significantly different between PDCoV- and PEDV-inoculated groups and PEDV- and coinoculated group at 3 DPI in the distal jejunum. The VH:CD ratios between single- and co-inoculated groups were significantly different at 5 DPI. The VH:CD ratio of the PDCoV-inoculated group was the highest, followed by that of the PEDV-inoculated group, and that of the co-inoculated group was the lowest. The VH:CD ratio of the PDCoV-inoculated group significantly increased at 5 dpi compared to 3 dpi. Meanwhile, the VH:CD ratio of the co-inoculated group decreased significantly at 5 dpi than 3 dpi.

In the duodenum, the VH:CD ratio of the co-inoculated group was significantly lowest compared to both single-inoculated groups at 3 and 5 dpi. The VH:CD ratio of the PDCoV-inoculated group was significantly higher than the PEDV-inoculated group at 5 dpi, but there was no difference at 3 dpi. It is interesting to note that the VH:CD ratio of the PDCoV-inoculated group was significantly higher at 5 dpi than 3 dpi.

In the ileum, the VH:CD ratio of both single-inoculated groups did not present a significant difference but was significantly higher than the co-inoculated group at 3 dpi. At 5 dpi, the VH:CD ratio of the PDCoV-inoculated group was significantly higher than 3 dpi and showed a significantly higher ratio than the PEDV- and co-inoculated group. However, the VH:CD ratio between the PEDV- and co-inoculated groups did not show significant differences.

### PDCoV was detected in both crypts and villous enterocytes in the co-inoculated group but only detected in villous enterocytes in the single-inoculated group

**Figure 4:**
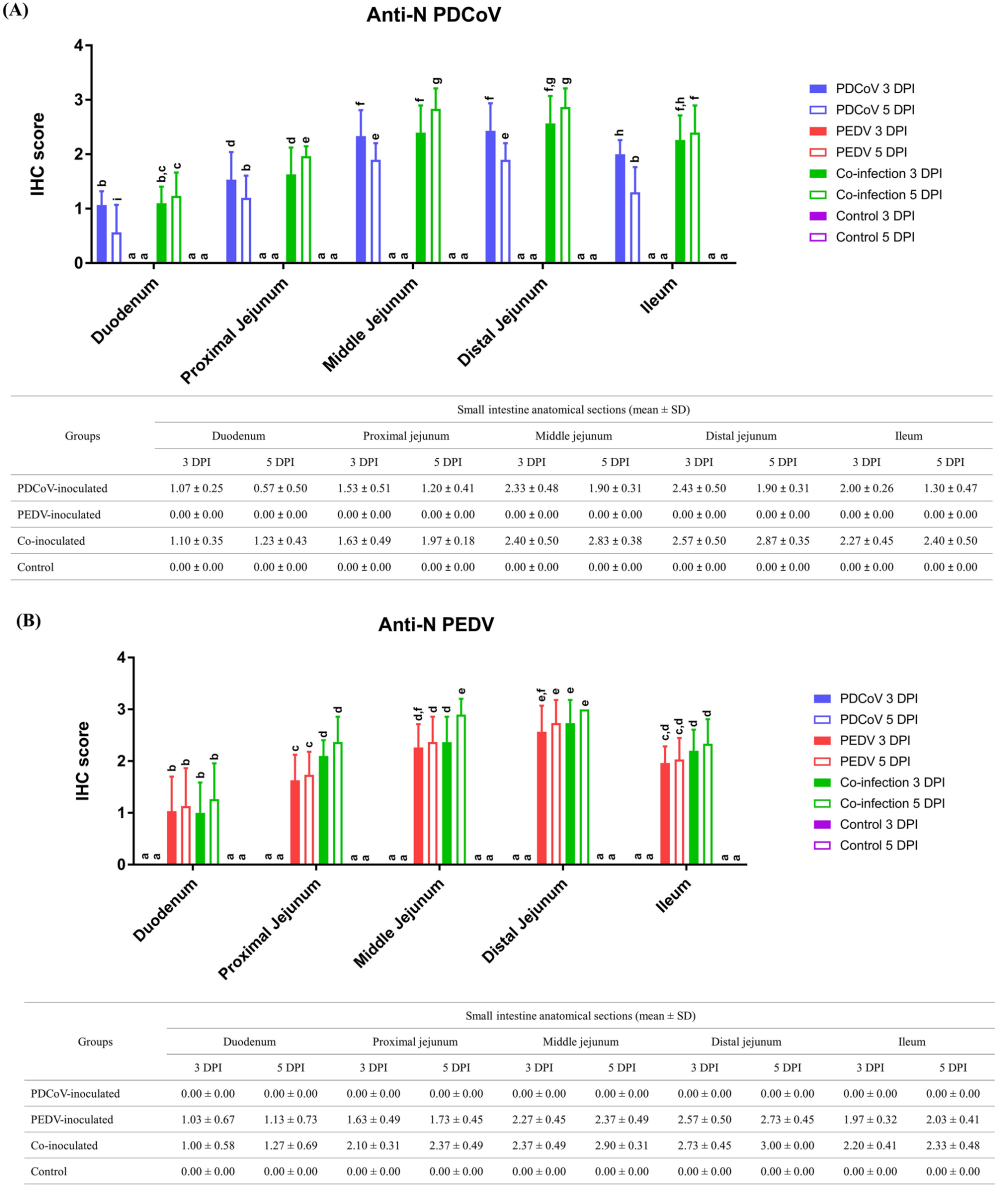
IHC scores of PDCoV (**A**) and PEDV (**B**) antigens in small intestinal enterocytes. Blue, red, green, and purple bars represent PDCoV-inoculated, PEDV-inoculated, co-inoculated, and control groups. Solid and open bars represent 3- and 5-days post-inoculation (dpi), respectively. Different lower-case letters indicate significant differences between each group, each dpi, and each anatomical section of the small intestine (*p* < 0.05). Values of mean ± standard deviation (SD) of each group, and each dpi are presented in a table under the graph.

The PEDV and PDCoV score IHC detection in 5 anatomical regions of the small intestine are shown in Fig. 4. PDCoV and PEDV antigen was detected by IHC in all regions of the small intestine. The PDCoV-inoculated group showed the highest IHC scores in the middle and distal jejunum, followed by the ileum, proximal jejunum, and duodenum, respectively (Fig. 4A). The PEDV-inoculated group showed the highest IHC score in the distal jejunum, followed by the middle jejunum, ileum, proximal jejunum, and duodenum, respectively (Fig. 4B). The co-inoculated group showed the highest IHC scores for both antigens in the middle and distal jejunum at 5 DPI.

In the duodenum, distal jejunum, and ileum, the PEDV-antigen IHC scores on the PEDV-inoculated group and PEDV- and PDCoV-antigen IHC score on the co-inoculated group was not significantly different between 3 and 5 dpi. However, the PDCoV-antigen IHC score in the PDCoV-inoculated group was significantly lower at 5 dpi than 3 dpi (Fig. 4A).

In the proximal jejunum, the level of PDCoV-antigen IHC score on the PDCoV-inoculated group decreased significantly from 3 to 5 dpi (Fig. 4A). No significant differences were observed in the PEDV-antigen IHC score in the single homologous inoculation group from 3 to 5 dpi. The levels of both PEDV- and PDCoV-antigen IHC score in the co-inoculated group was significantly higher compared with each single inoculated group (Fig. 4B).

In the middle jejunum, PEDV, and PDCoV, antigen IHC scores were similar to the proximal jejunum. The PDCoV-antigen IHC scores in the PDCoV-inoculated group decreased significantly from 3 to 5 dpi (Fig. 4A). The co-inoculated group showed the highest levels of both viral antigens significantly compared with each single inoculated group.

In the distal jejunum, the PDCoV-antigen IHC scores in the PDCoV-inoculated group decreased significantly from 3 to 5 dpi (Fig. 4A). The showed the significantly highest level of The PDCoV-antigen IHC score was significantly higher in the co-inoculated group compared to the single PDCoV-inoculated at 5 dpi. The PEDV-antigen IHC score between PEDV- and co-inoculated groups had no significant differences at 3 and 5 dpi.

**Figure 5:**
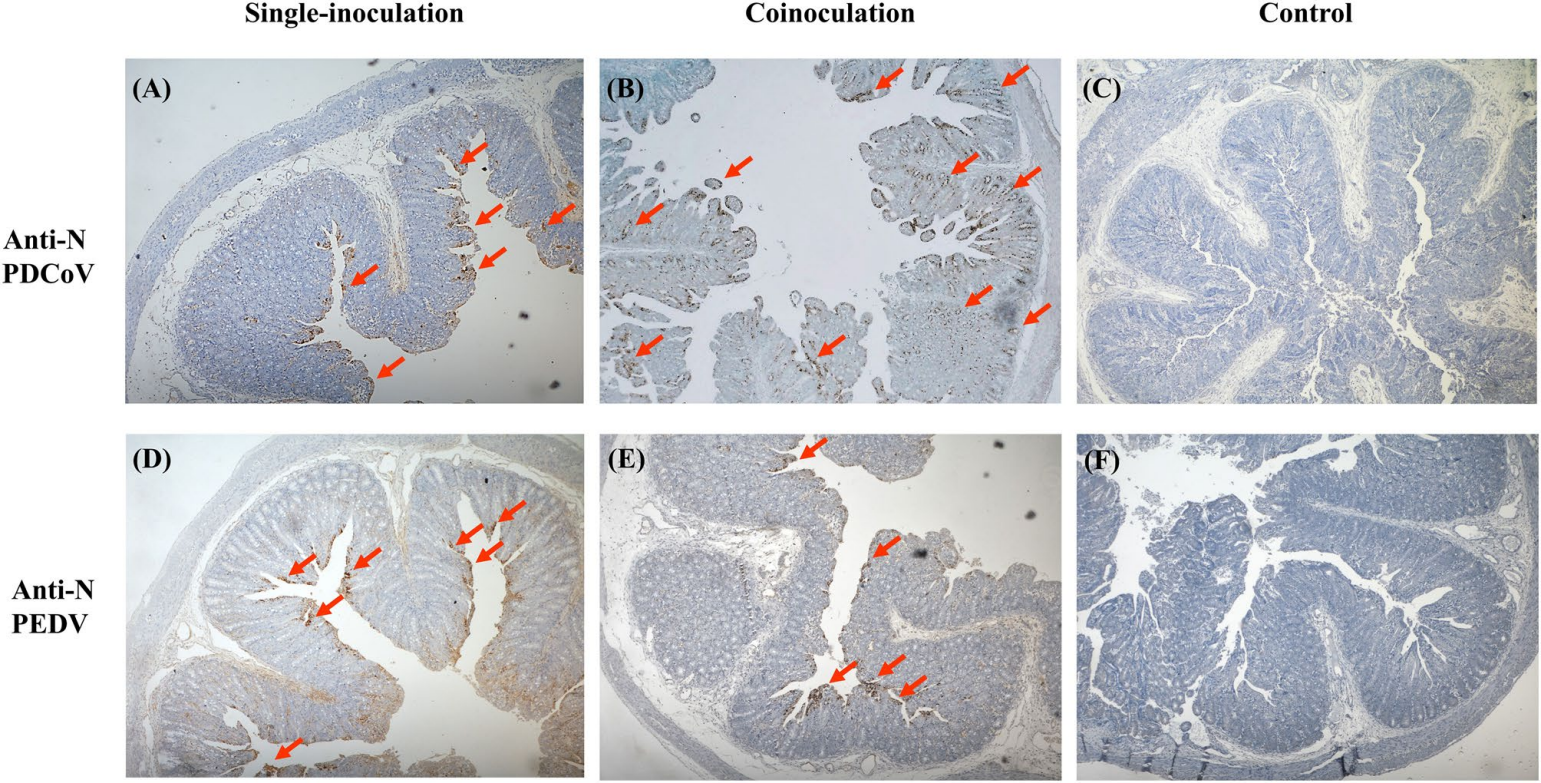
Immunohistochemistry (IHC) analysis (20X magnification) of small intestines of PDCoV-infected (**A**), PEDV-infected (**D**), co-infected (**B** and **E**), and control groups (**C** and **F**). Panels A to C were stained with anti-N PDCoV antibody. Panels D to F were stained with anti-N PEDV antibody. Red arrows represent enterocytes with positive signals for each virus.

Interestingly, PDCoV antigen was detected only in villous enterocytes in the single-inoculated group (Fig. 5A) but was detected in both crypts and villous enterocytes of all small intestinal regions, especially in the middle and distal jejunum in the co-inoculated group (Fig. 5B). PEDV antigen was detected only in villi enterocytes either in single- (Fig. 5D) or co-inoculation (Fig. 5E) cases. No cross staining was detected. Negative controls were negative for both PDCoV (Fig. 5C) and PEDV IHCs (Fig. 5F).

### Coinoculation of PDCoV and PEDV early up-regulated IFN-α and IL12

**Figure 6:**
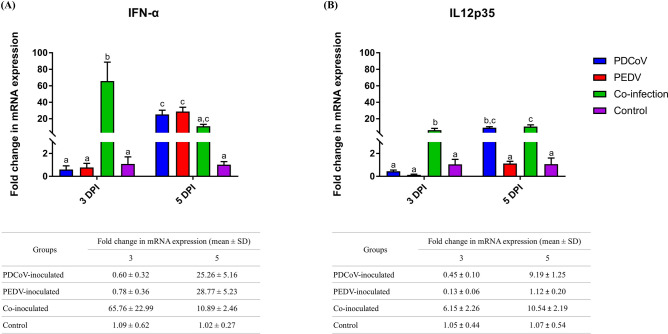
mRNA expression of proinflammatory cytokine genes on the intestinal mucosa of neonatal pigs. The mRNA levels of IFN-α (**A**) and IL12 (**B**) were determined using qPCR. Glyceraldehyde-3-phosphate dehydrogenase (GAPDH) and beta-actin were used as an internal control to normalize changes in specific gene expressions. The results were presented as fold changes relative to the control animals. Different lower-case letters indicate significant differences between each group and each dpi (*p* < 0.05). Values of mean ± standard deviation (SD) of each group, and each dpi are presented in a table under the graph.

The expressions of IFN-α and IL12 genes are presented in Fig. 6. Significant upregulation of IFN-α and IL12 genes and only IL12 gene were detected at 3 and 5 dpi, respectively, in the co-inoculated group compared to that of either single inoculated group (Fig. 6B). PDCoV single-inoculation had no regulatory effect on cytokines by 3 dpi; however, IFN-α and IL12 were significantly up-regulated compared with the negative control by 5 dpi. Similar to PDCoV, PEDV-single-inoculation had no genomic regulatory effect on IFN-α and IL12 by 3 dpi. However, PEDV single-inoculation significantly up-regulated IFN-α gene expression by 5 dpi compared to the negative control group (Fig. 6A).

## Discussion

PEDV and PDCoV have been become endemic in the SEA region since their first emergence in 2007 and 2013, respectively^13, 26^. Sporadic outbreaks of diarrhea associated with porcine enteric coronaviruses are routinely observed, and both PEDV and PDCoV have been simultaneously detected^22, 26–29^. It leads to the speculation that the co-infection of these 2 viruses could potentially enhance the severity of enteric clinical cases. The pathogenicity of PEDV and PDCoV has been intensively investigated^4, 37, 38^. However, co-infection studies with these two viruses have not been reported elsewhere. Therefore, the study conducted herein was designed to investigate the severity of enteric clinical disease following PEDV and PDCoV co-infection. This study’s experimental design includes four days-old pigs to represent the field situation in which pigs at this age are more susceptible to both PEDV and PDCoV infection than older pigs. The clinical disease severity difference was evaluated through diarrhea severity, viral shedding, intestinal VH:CD ratio, and viral distribution in the small intestine. The results of this study demonstrated that PEDV-PDCoV co-infection increased the severity of enteric clinical signs, demonstrated by a significant increase in fecal score, prolonged viral shedding, and a significant villi-shortening in multiple anatomical regions of the small intestine compared with pigs single-inoculated either with PEDV or PDCoV. The antigen detection by IHC demonstrated evidence suggest that the co-infection enhances tissue tropism. Notably, in single-inoculated groups, PEDV and PDCoV antigens were detected only in villous enterocytes. However, in the co-inoculated group, PDCoV antigens were detected in both villous enterocytes and crypts.

In contrast, PEDV antigens were only detected in villous enterocytes similar to that of the single infection. It is also noteworthy that pigs in the single PDCoV group displayed a milder disease severity than the PEDV- and co-infection groups but exhibited signs of recovery by 5 dpi. This is in contrast with pigs singly inoculated with either PEDV or PDCoV. The single PDCoV group’s recovery signs were supported by histopathological and IHC findings, in which higher VH:CD ratio and lower PDCoV antigen in tissues were observed.

Diarrhea was first observed in all inoculated groups, either single- or co-inoculation at 1 dpi, and the fecal score reached the highest level at 3 dpi. However, the co-inoculated group displayed the highest fecal score compared to the single-inoculated group. This evidence suggests that the onset of clinical disease is not different between PEDV and PDCoV infection, either alone or in co-infection cases, but that it induces a more severe clinical disease.

The results of onset of clinical signs, diarrhea severity, and viral shedding in fecal samples in a single infected group, either PEDV or PDCoV are in agreement with those reported previously^4, 36, 41–44^. However, the ages of challenged pigs, clusters, and doses of PDCoV were different. Previous PDCoV studies using 2 different age groups of pigs, including weaned and newborn pigs, and a PDCoV isolate (PDCoV CHN-GD-2016) in China cluster report the onset of clinical diarrhea early as 1 dpi^36, 44^. Diarrhea severity and fecal shedding were highest at 3 dpi and continuously reduced after that. The study involving 4 different US PDCoV isolates (PDCoV OH-FD22, OH-FD100, Ohio CVM1, or MI strain) also reported similar diarrhea manifestation findings at 21–24 h post-inoculation (HPI)^4, 42^. Similar to PDCoV, previous PEDV studies report that the onset of clinical diarrhea is detected as early as 1 dpi following the PEDV isolate US PEDV PC21A challenge in 9-day-old piglets^41^. Fecal consistency scores significantly increased and remained at the highest level, from 3 to 5 dpi^41^. In another study, fecal consistency score and PEDV shedding were significantly increased from 1 to 4 dpi^43^.

Pigs in PEDV- and co-inoculated groups exhibited severe diarrhea from 3 to 5 dpi, and there were no signs of recovery. However, pigs in the PDCoV-inoculated group displayed signs of recovery. Viral shedding also followed the same pattern of variation of the fecal score. The co-inoculated group had the highest virus levels at 5 dpi compared to single infection groups. The levels of both viruses increased from 3 to 5 dpi in the co-inoculated group. In contrast, the shedding pattern of PEDV remained similar from 3 to 5 dpi, while the levels of PDCoV continuously decreased from 3 to 5 dpi in the single-inoculated groups.

Macroscopic changes consistent with viral infection characterized by thin and translucent intestinal walls and distended by fluid accumulation were observed in all inoculated piglets. These macroscopic lesions were indistinguishable between the single- and co-inoculated groups. Although general microscopic findings, characterized by villous shortening and blunting, VH:CD ratios were similar; the severity differed between groups. VH:CD ratio results are in agreement with fecal score and viral shedding findings in which the co-infected group was more severe than single inoculated groups, showing significantly villus attenuation (lower VH:CD ratio in the jejunum). These histological changes also support the co-infection synergistic effect since no differences in VH:CD ratio in the single PEDV- and PDCoV-inoculated groups. In addition, to the viral shedding and VH:CD ratio results, the IHC score of the single-inoculated groups showed that PEDV-antigen rate detection was consistent during the study, while the amount of PDCoV-antigen decline overtime suggesting PDCoV has the highest clearance rate in all anatomical regions of small intestinal regions. However, viral clearance seems to be impaired during co-infection challenge since neither antigen rate decreased over time, especially in the middle jejunum. Thus, the findings of the present study suggest that PEDV and PDCoV co-infection might affect host cellular factors that impair viral clearance and/or increase the number of target cells each virus can infect. However, further studies are necessary to understand this potential mechanism further.

Notably, the study unveiled exciting findings regarding the cell tropism in the small intestine for each enteric coronavirus. In co-infected pigs, PEDV antigen was detected only in villous enterocytes. Simultaneously, PDCoV-antigen was detected in the intestinal villous and crypt enterocytes, especially in the middle and distal jejunum. In contrast, we found that both PEDV and PDCoV antigens were detected only in villous enterocytes when infected individually. The detection of PEDV and PDCoV antigens only in the villous enterocytes in a single infection observed in this study is in agreement with previous reports^1, 4, 36, 37, 41–46^. A previous study reported that PDCoV antigen was rarely detected in crypt epithelial cells of the jejunum and ileum of a pig infected with the PDCoV OH-FD100 strain^47^. However, in this study, PDCoV antigen was detected in crypts enterocytes in the co-infected group, while in the PDCoV-inoculated group, PDCoV antigen was detected only in villous enterocytes. These findings suggest that PEDV-PDCoV co-infection increased the cell tropism of PDCoV. From these results, we hypothesize that, in the co-inoculated group, (1) PEDV has restricted cell tropism, and/or (2) PDCoV has the ability to expand cell tropism due to differential receptors expression in the crypt enterocytes during co-infection (3), and co-infection might impair viral clearance due to exacerbated modulation of inflammatory and pro-inflammatory cytokines genes. These results suggest that co-infection enhances PDCoV, but not PEDV, intestinal fitness. These hypotheses are also supported by previous reports that PEDV has been found only in pigs^48^, while PDCoV has a broad species tropism, including chickens and cattle^49, 50^. Further studies are necessary to confirm these hypotheses.

Previous field studies reported that the severity and mortality of PDCoV outbreaks are lower compared to PEDV outbreaks^4, 5^. However, there are no previous reports comparing the virulence of these two viruses. The results of this study comparatively demonstrated that PDCoV is less virulent than PEDV, supported by lower diarrhea severity and faster clinical recovery at the end of the study. Although the intestinal lesions induced either PEDV or PDCoV were indistinguishable, the severity of the VH:CD ratio demonstrated also supports these differences in pathogenicity supporting the observed clinical signs. Besides, in PDCoV infected animals, the clinical recovery is supported by the higher VH:CD ratio at 5 dpi compared to 3dpi. Other mechanisms involving lower virulence of PDCoV compared to PEDV require further investigation. Although several factors, including mucosal cellular immunity or viral-regulatory effect in the anti- and proinflammatory response, should be evaluated.

Thus expression of IFN-α and IL12 was evaluated in the small intestinal mucosa either in single- or co-inoculated animals. IFN-α is a pluripotent inflammatory cytokine naturally induced by viral infections. IL12 is an innate cytokine produced by macrophages and dendritic cells that can be stimulated during viral infections. However, PEDV and PDCoV were previously reported to antagonize the production of type I IFN and cytokines in vitro^51–55^. The expressions of IFN-α and IL12 have been evaluated in vivo in a single PEDV or PDCoV infection models^35, 36, 56, 57^. Thus, in vivo studies showed that IL12 and type I IFNs were induced at 3 days after infected with PDCoV and, PEDV up-regulates IFN-α and IL12 expression after 16 post-inoculation hours to 3 dpi^35, 56, 57^. Results in this study are consistent with previous in vivo reports; however, regulatory levels of IFN-α and IL12 in the PDCoV-inoculated group and IFN-α in the PEDV-inoculated group were lower. In this study, there was not a detectable modulatory effect of IL12 in the PEDV-inoculated group. The difference in IL12 modulatory effect between this study and other previously reported could be associated with the timing of evaluation of modulatory effects amongst others, infectious dose, and viral strain. It has been reported that differences in viral strains might lead to differences in innate immune response modulation^35, 36, 56, 58^.

Interestingly, PEDV and PDCoV co-infection induced an earlier positive modulatory on IFN-α and IL12 expression than single-inoculation. Thus from these results, we hypothesize that PEDV and PDCoV co-infection might have a synergistic effect similar to co-infection of another swine viruses such as porcine circovirus type 2 (PCV2), swine influenza virus (SIV), and porcine reproductive and respiratory syndrome virus (PRRSV) leading to IFN and proinflammatory cytokine changes^59–61^. Moreover, the early positive modulatory effect on IFN-α and IL12 gene expression in the co-inoculation group might be among the leading causes of more severe disease than a single-inoculation. This modulatory effect exerted by a viral co-infection might impair viral clearance exacerbating the clinical outcome. Due to the important role of IFN-α and IL12 in the innate immune system working as the first line of defense during viral infection, the stimulation of the innate signaling pathway molecules and cytokines on the small intestinal mucosa of neonatal piglets during PDCoV and/or PEDV should be further evaluated.

## Conclusion

In conclusion, this study is the first report of PDCoV and PEDV co-inoculation in neonatal piglets. Our findings suggest that PEDV and PEDCoV’s co-infection aggravates the disease severity due to increased viral shedding, reduction of VH:CD ratio in different anatomic regions of the small intestine, and increased levels of viral infection in small intestinal enterocytes. In addition, PDCoV increases the range of cellular targets in the intestinal mucosa during co-infection with PEDV. We hypothesize that PEDV might have a better and adapted affinity for the villous enterocytes than PDCoV, leading to PDCoV infection of crypt enterocytes instead in the co-infection model. However, this hypothesis needs to be further evaluated. Moreover, the earlier induction of IFN-α and IL12 expression in the co-infection group might be one of the leading causes of more severe disease than a single-inoculation.
